# Incidence, demographics and rehabilitation after digital nerve injury: A population-based study of 1004 adult patients in Sweden

**DOI:** 10.1371/journal.pone.0283907

**Published:** 2023-04-07

**Authors:** Linda Evertsson, Carin Carlsson, Christina Turesson, Melih Selcuk Ezer, Marianne Arner, Cecilia Mellstrand Navarro

**Affiliations:** 1 Department of Clinical Science and Education, Karolinska Institutet, Stockholm, Sweden; 2 Department of Hand Surgery, Sodersjukhuset Hospital, Stockholm, Sweden; 3 Division of Prevention, Department of Health, Medicine and Caring Sciences, Rehabilitation and Community Medicine, Linkoping University, Norrkoping, Sweden; BG Trauma Center Ludwigshafen, GERMANY

## Abstract

**Objective:**

The main objective of this study was to describe the epidemiology of surgically repaired digital nerve injuries in a Swedish population. Secondary objectives were to describe the demographics of the patient population, injury characteristics, post-operative care and rehabilitation.

**Methods:**

From 2012 to 2018, 1004 patients with a surgically repaired digital nerve injury resident in the Stockholm region were identified in the Swedish national quality registry for hand surgery and all medical records were thoroughly reviewed.

**Results:**

The incidence rate was 8.3 per 100.000 person-years and these injuries were more common in men than women. The median age at the time of injury was 37 years and a sharp cut was the most common mechanism of injury. Injuries were equally distributed over weekdays and the year, but surgery was most often performed on Mondays. There were no differences in treatment and rehabilitation regimens between sexes, except women were more likely than men to be operated within three days from injury. Timing and content of rehabilitation varied largely between individuals. One third of patients did not receive any sensory relearning and sensory assessment was performed in only 7%.

**Conclusion:**

The epidemiology shows no major changes over the last decade. However, we found a large individual variation in follow up visits, rehabilitation content and assessments indicating large differences in consumption of health care resources. Our findings expose the need to further improve and evaluate rehabilitation regimens after digital nerve injury.

## Introduction

Digital nerve injuries are the most common upper limb peripheral nerve injuries [1–3], with a reported incidence of 6.2/100 000 inhabitants in a Swedish population [1]. The injury mechanism is often trivial, such as a knife cut during household activities or a cut from broken glass. For the individual, the injury results in immediate impaired sensory function of the fingertip. Despite the minor nature of the injury, some patients develop persistent disability and pain. Considerable health care resources are invested in these patients, but there are few large studies describing incidence, complications, treatments [4], contents of rehabilitation [5–7] and results.

Early rehabilitation interventions have been proven beneficial for recovery after major nerve injuries [8, 9], with early sensory relearning starting immediately after injury and before re-innervation of the axons in the hand occurs [10, 11]. However, the use of rehabilitation regimens and their impact on clinical outcomes after digital nerve injuries has not yet been thoroughly investigated.

Our primary objective was to describe the epidemiology of surgically repaired digital nerve injuries in the Stockholm region in Sweden. Secondary objectives were to describe demographics of the patient population, injury characteristics, post-operative care and rehabilitation methods.

An understanding of the epidemiology, description of the patient and care provided, could be of value to achieve a more equal care.

## Methods

This study is a retrospective population-based cohort study of adult patients treated with surgical repair of a digital nerve injury in Stockholm, Sweden between January 2012 and December 2018. The Stockholm region mainly consists of an urban population, with approximately 2 million inhabitants. Care for nerve injuries in the Stockholm area is centralised to the department of hand surgery at Södersjukhuset Hospital which provides specialised surgical treatment and in-house rehabilitation. Patients with digital nerve injuries are primarily attended in the emergency department in any hospital of the Stockholm region. A referral takes place on the day of injury when possible and the patient is examined in the department of hand surgery at Södersjukhuset Hospital on the same or next available weekday. Surgery is performed as soon as resources are available in the operating theatre. The STROBE guidelines for reporting observational studies were used as guidance.

The research was performed according to the Declaration of Helsinki. The Swedish National Quality Registry for Hand Surgery (HAKIR) was used in the study. Entering the HAKIR registry requires detailed information to all participants with offered opt-out from the registry if so desired, following Swedish legislation. The need for written consent for use of registry data and medical journal search used for this study was waived by the Swedish Ethical Review Authority (Dnr 2019–05984 and 2021–01519). The study was registered in Clinicaltrials.gov. (NCT05269719). All data for analysis were pseudonymized. Data is only presented at group level and no individuals can be identified in the study.

### Patients

**Fig 1:**
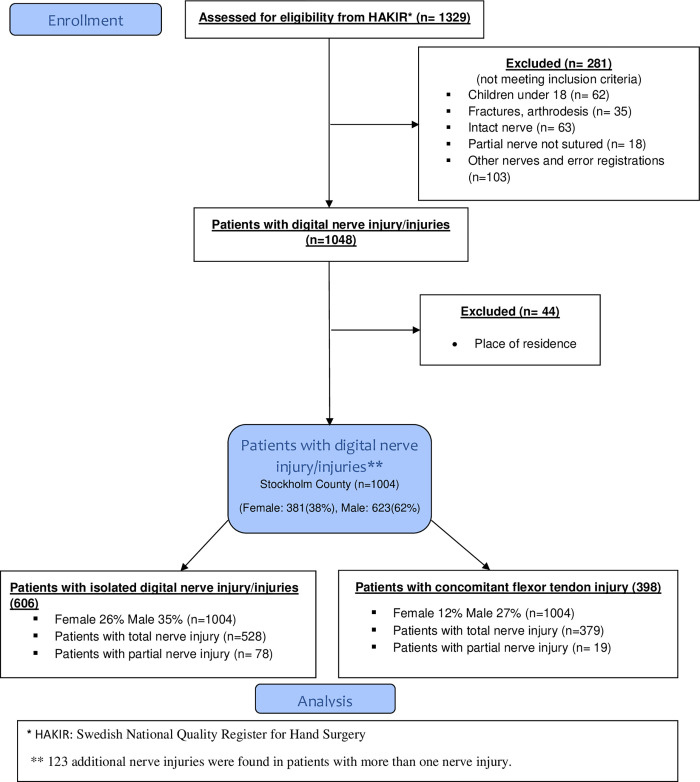
Flowchart of inclusion of digital nerve injury in Stockholm, Sweden 2012–2018.

Patients were identified in the Swedish national quality registry for hand surgery [12] using the following inclusion criteria: diagnostic codes (ICD-10) for a digital nerve injury in the thumb (S64.3) or finger (S64.4) in combination with the surgical code (KKÅ97) [13] for peripheral nerve suture (ACB29). In total, 1329 patients were identified. Exclusion criteria were concomitant skeletal injury, amputations and severe soft tissue injuries if microvascular reconstruction was mandated. Digital nerve injury with concomitant injury to digital arteries were included if circulation was not insufficient. Patients residing outside the Stockholm region and children aged <18 years were also excluded. After a thorough review of all medical records, 1004 patients were included for final analysis (Fig 1). The study population was divided into two groups: patients with an isolated digital nerve injury and those with a concomitant flexor tendon injury.

### Surgical treatment and rehabilitation

The digital nerves were sutured according to the preference of the treating surgeon, and the standard procedure was three epineural sutures (8–0 or 9–0 nylon (S & T ®)) under loupe magnification. Concomitant flexor tendon injuries were also repaired according to the preferences of the treating surgeon, most commonly with two loop sutures (four-strand core sutures) and a peritendinous polydioxanone suture. All surgical repairs were performed or supervised by surgeons with Level III expertise [14]. Patients with concomitant flexor tendon injuries were immobilised in a cast for 4 weeks and isolated digital nerve injuries in a cast or a stabile dressing for 2–3 weeks [15], according to local standards. The surgeon decided whether referral for rehabilitation was indicated and the rehabilitation was conducted at the discretion of the therapist.

### Data sources and data collection

The HAKIR registry provided data on date of birth, sex, injured hand, date of injury and surgery and coding for diagnoses and surgical procedures. All subjects were fully informed and offered opt-out from registration at inclusion in HAKIR according to regulations for quality registries. All medical records were thoroughly reviewed by the authors (LE, CC, MA or MS) to collect all other data for the study (Table 1).

**Table 1:** Patient and injury data collected from medical records in Stockholm, Sweden, 2012–2018.

Category	Variable	Subgroups
Patient	Smoking	Non-smoker, previous smoker, smoker or unknown
	Profession	Non-workers^1^, light, moderate or heavy manual workload
Injury	Location	Palm, proximal or middle phalanx
	Mechanism	Sharp, saw, crush or other
	Extent of injury	Total or partial injury
	Presence of a concomitant flexor tendon injury or not	Isolated nerve injury or nerve injury with concomitant flexor tendon injury
	Day of the week and month	Monday to Sunday, January to December
Complications	Infection	Yes or no
	Re-rupture of a repaired flexor tendon	Yes or no
	Neuropathic pain	Yes^2^ or no
Rehabilitation interventions	Time between injury and interventions	Number of days
	Sensory assessments	Yes^3^ or no
	Sensory relearning	Early sensory relearning, traditional sensory re-education or non-structural exercises^4^
	Desensitisation	Yes^5^ or no
	Cold intolerance treatment	Yes^6^ or no
	ADL activity exercises	Yes^7^ or no
	Motion exercises	Yes or no
	Splinting	Yes^8^ or no
	Scar treatment	Yes^9^ or no
	Oedema treatment	Yes^10^ or no
	Pain treatment	Yes^11^ or no
Health care use	Number of visits to different health care professionals^12^	Surgeon, physiotherapist, occupational therapist, nurse or social worker
	Sick leave	Number of days

^1^Retired patients and students were classified as non-workers.

^2^Neuropathic pain was classified as present if mentioned in the medical records or if ICD-10 code M79.2 was found.

^3^Documented in medical records as Semmes Weinstein Monofilaments and/or Static 2 Point Discrimination.

^4^Defined as interventions in which the patient learns how to improve the interpretation of sensation by using the hand in daily activities.

^5^Desensitising treatments mainly comprised submerge the hand into beans, rice and other materials.

^6^Identified in medical records as cold management information or heat products.

^7^Documented in medical records as the involvement of daily use of the hand and encouragement to use the injured finger.

^8^Identified as a soft splint for protection, splint to improve motion or splint to prevent contractures.

^9^Documented scar treatment was classified as present if the use of silicone gels, sheets or scar immobilization was mentioned.

^10^Involved elevation exercises, oedema gloves, external wrapping and pressure garments.

^11^Information, TENS (transcutaneous electrical nerve stimulation) or local anesthesia dressings.

^12^Phone calls and standardized follow-up measures for flexor tendon injuries were excluded.

### Statistical analysis

Incidence was calculated as the number of injured patients divided by the population at risk collected from Statistics Sweden [16] and presented as incidence per 100 000 person-years with a 95% confidence interval (CI) (95% Confidence Intervals for a Rate [17]). Numerical data were tested for normality using Shapiro Wilk’s test and histograms. None of the data was normally distributed. Accordingly, all central tendencies were presented as medians with a corresponding interquartile range (IQR). The Mann-Whitney U test was used to compare groups. Categorical variables were presented as numbers and proportions, expressed in percentages and presented with 95% CIs. The chi-square test was performed to determine group differences. A logistic regression analysis was conducted to analyse time from injury to operation (dichotomised as performed within 3 days or later) controlled for age, sex, concomitant flexor tendon injury, and type of injury mechanism. Crude and adjusted analyses are presented as odds ratios (ORs) with corresponding 95% CIs. We used the statistical software SPSS® version 28.

## Results

### Patients

A total of 1004 adult patients were included during a 7-year study period. Demographics are presented in Table 2.

**Table 2:** Demographics of 1004 patients treated surgically for a digital nerve injury in Stockholm, Sweden, 2012–2018.

		N	%
**Sex**	Male	623	(62)
	Female	381	(38)
**Occupation**	Non-worker	164	(16)
	Light manual workload	351	(35)
	Moderate manualworkload	283	(28)
	Heavy manual workload	170	(17)
	Missing	36	(4)
**Smoking**	Non-smoker	521	(52)
	Smoker	185	(18)
	Previous smoker	71	(7)
	Missing	226	(23)
**Injured hand**	Right	336	(34)
	Left	666	(66)
	Bilateral	2	(0.2)
**Type of injury **	Total nerve injury	907	(90)
	Partial injury	97	(10)
	Concomitant flexor tendon injury	398	(40)
	Isolated nerve injury	606	(60)
**Type of injurymechanism**	Sharp	868	(86)
	Saw	69	(7)
	Crush injury	7	(1)
	Other	60	(6)
**Complications**	Infection	38	(4)
	Neuropathic pain	19	(2)
	Tendonre-rupture	49	(5)

N = numbers.

() = percentage.

**Fig 2:**
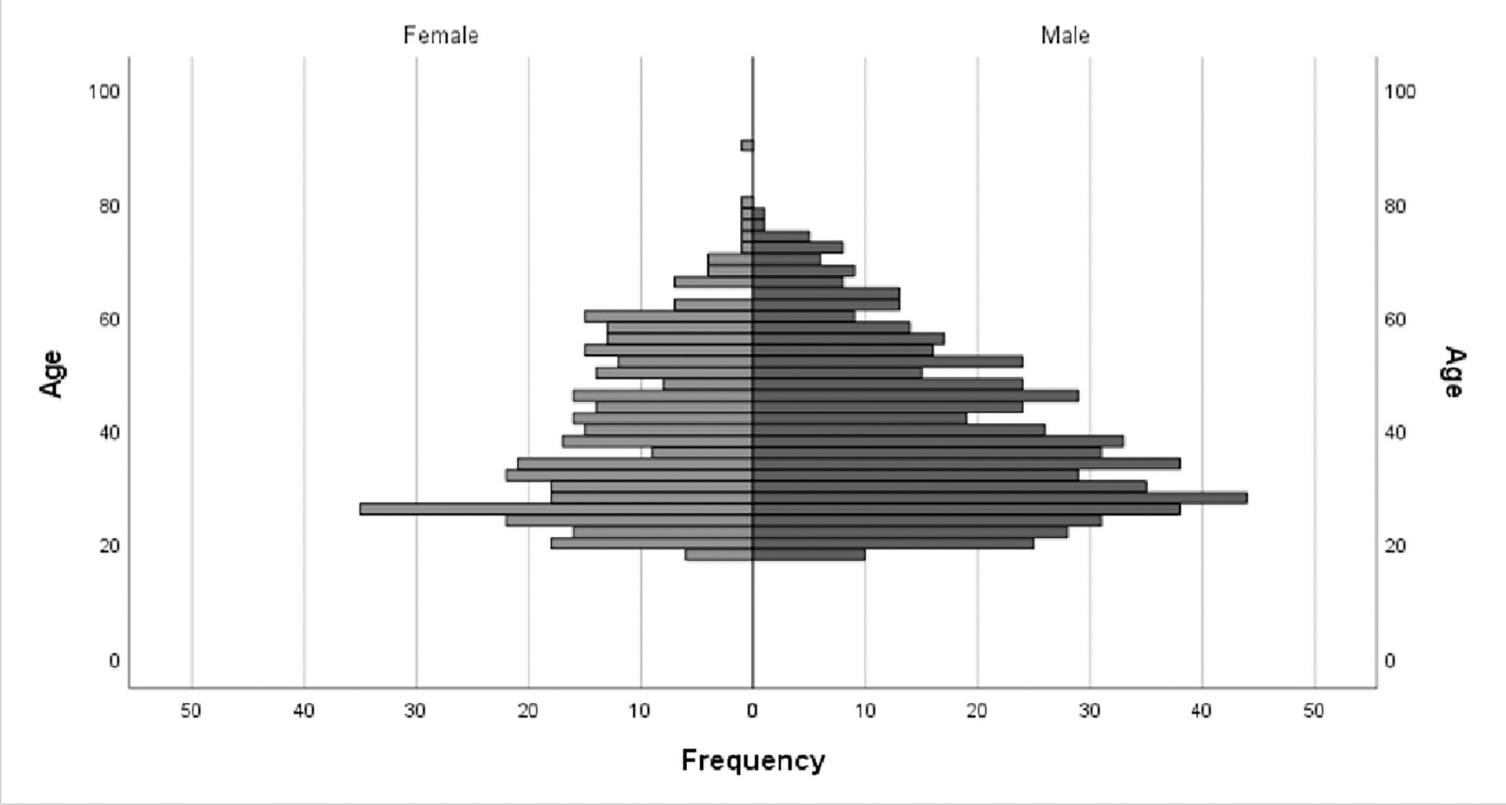
Pyramid histogram showing age and sex distribution.

The incidence rate of surgical repair of an isolated digital nerve injury was 8.3 per 100 000 person-years. Age at injury ranged from 18–90 years, with a median age of 37. Age distribution, skewed towards younger age, did not differ between female (n = 381; median age 38 years) and male patients (n = 623; median age 37 years) (Fig 2).

### Injury

**Fig 3:**
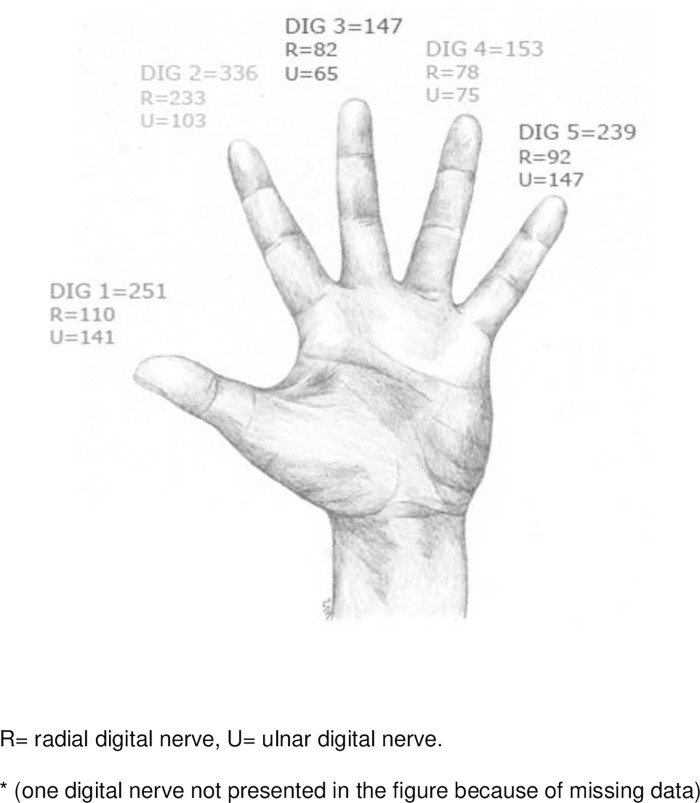
Distribution of 1127* digital nerve injuries in 1004 patients.

**Fig 4:**
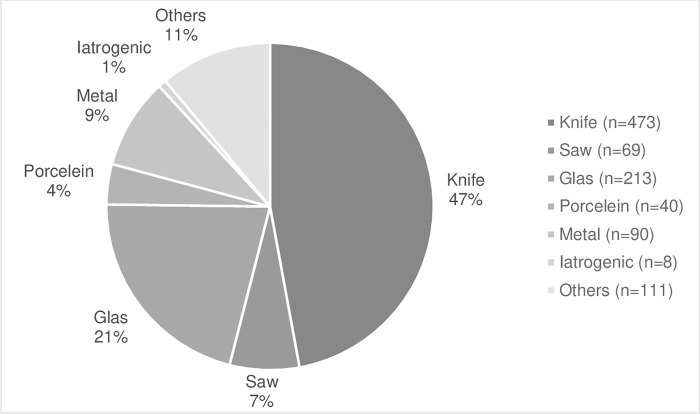
Pie chart presenting cause of injury.

A number of 1127 separate nerve injuries in 1004 patients were analysed. One-hundred and fifteen patients (11%) were diagnosed with at least two nerve injuries and three with multiple nerve injuries. Partial nerve injuries were found in 99 patients (10%). 30% of the injuries were to the index finger, and the most common level was the proximal phalanx (54%) (Fig 3). Concomitant flexor tendon injuries occurred in 398 patients (40%) and were more common in men (69%, n = 276) than in women (31%, n = 122) (Fig 1). Most injuries (86%, n = 868) were from sharp cuts. The most common cause of injury was a knife (47%, n = 473), followed by glass (21%, n = 213). Injury when the knife slips while splitting an avocado accounted for 6% (n = 61) of all injuries (Fig 4).

**Fig 5:**
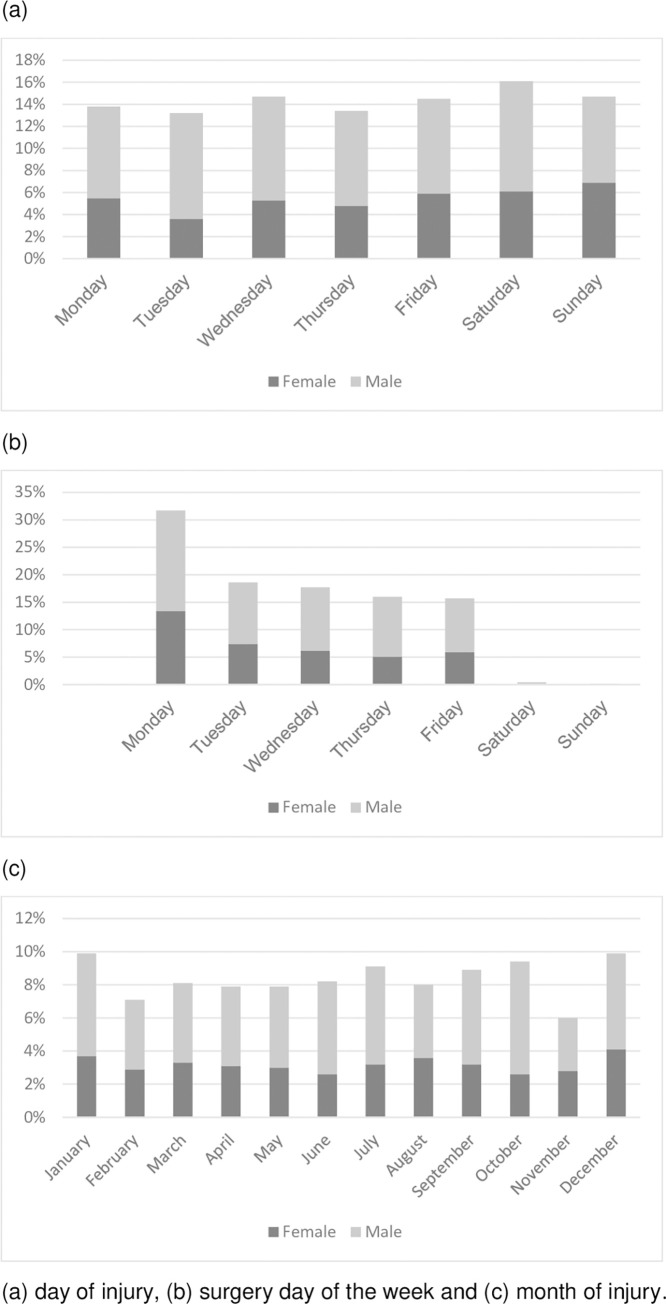
Bar chart presenting timing.

Injuries occurred on all days of the week and no statistically significant differences were noted over the week (Fig 5A). However, surgical repairs were most often performed on Mondays (Fig 5B). Injuries were evenly distributed over the months of the year (Fig 5C).

### Time between injury and surgery

The median time from injury to surgery was 2 days (IQR 3, range 0–169). The median time for women was 3 days (IQR 4, range 0–169) and 2 days for men (IQR 3, range 0–90). However, when analysed in a logistic regression controlling for age and type of injury, women with digital nerve injuries were more likely than men to be treated surgically within 3 days of injury. This difference was statistically significant in the crude and adjusted analyses (Table 3).

**Table 3:** Logistic regression analysing time from digital nerve injury to surgery adjusted for sex, type of injury and mechanism in 1004 patients treated surgically for a digital nerve injury in Stockholm, Sweden, 2012–2018.

				Crude analysis			Adjusted analysis		
		n	%	OR	95%CI	P-value	OR	95%CI	P-value
**Sex**	**Male**	623	62	ref			ref		
	**Female**	381	38	1.441	1.115–1.863	0.005	1.304	1.001–1.697	0.049
**Isolated nerve injury**	**Yes**	606	60	ref			ref		
	**No**	398	40	0.674	0.521–0.872	0.003	0.692	0.534–0.898	0.006
**Sharp injury**	**Yes**	868	86	ref			ref		
	**No**	136	14	0.595	0.407–0.870	0.007	0.634	0.430–0.935	0.021

N = numbers

OR = odds ratio

CI = confidence interval

Ref = reference category

P-value< 0.05 was considered statistically significant

### Complications

A post-operative complication was noted in 7% of the patients. The post-operative infection rate was 4% (n = 38). Neuropathic pain was reported in 2% (n = 19) of the patients.

### Sick leave

The study population had a median sick leave of 33 days (IQR 60, range 0–448). Patients with a concomitant flexor tendon injury had a significantly longer sick leave (86 days (IQR 64, range 0–448)) than patients with an isolated digital nerve injury (28 days (IQR 26, range 0–214)), p<0.001. Male patients had a significantly longer sick leave than female patients with 40 days (IQR 67, range 0–379) versus 30 days (IQR 38, range 0–448) (p<0.001).

### Care and rehabilitation

The number of visits to different health care professionals is presented in Table 4.

**Table 4:** Number of visits to health care personnel after surgical treatment for a digital nerve injury in Stockholm, Sweden, 2012–2018.

Injury		Physician	Occupational therapist	Physio therapist	Nurse	Social worker
**Isolated**	Women, N (IQR) *range*	1 (1) *0–9*	2 (2) *0–18*	1 (1) *0–10*	1 (1) *0–14*	0 (0) *0–9*
**nerve**	Men, N (IQR) *range*	1 (1) *0–7*	1 (2) *0–13*	1 (2) *0–8*	1 (1) *0–6*	0 (0) *0–2*
**injury**	Total	1 (1) *0–9*	1 (2) *0–18*	1 (2) *0–10*	1 (1) *0–14*	0 (0) *0–9*
**Concomitant tendon injury**	Women, N (IQR) *range*	3 (2) *0–8*	3 (4) *0–28*	6 (5) 0–22	1 (1) *0–8*	0 (0) *0–4*
	Men, N (IQR) *range*	3 (2) *0–18*	3 (5) *0–25*	6 (5) *0–22*	1 (2) *0–6*	0 (0) *0–7*
	Total	3(2) *0–18*	3 (5) *0–28*	6 (5) *0–22*	1 (2) *0–8*	0 (0) *0–7*

N = median numbers

IQR = interquartile range

Three-hundred and sixteen patients (52%) with an isolated digital nerve injury and 76 (19%) patients with a concomitant flexor tendon injury did not have a postoperative visit to a physician. No difference between the sexes was seen. Most patients (92%, n = 927) visited an occupational or physical therapist. The most common rehabilitation intervention was motion exercises (82%, n = 825), followed by sensory relearning (67%, n = 677) (Table 5).

**Table 5:** Number of patients subject to rehabilitation after surgical treatment for a digital nerve injury in Stockholm, Sweden, 2012–2018.

		Assessment	Intervention						
Injury		Sensory assessment	Sensory relearning	Desens itisation	Pain treatment	Cold intolerance treatment	Motion exercise	Activity exercise	Splints
**Isolated nerve injury**	Women	25 (10%)	187 (72%)	120 (46%)	35 (14%)	40 (16%)	199 (77%)	159 (61%)	72 (28%)
	Men	26 (8%)	243 (70%)	122 (35%)	25 (8%)	55 (16%)	256 (74%)	168 (48%)	96 (28%)
**Concomitant flexor tendon injury**	Women	8 (7%)	83 (68%)	50 (41%)	15 (12%)	18 (15%)	117 (96%)	98 (80%)	105 (86%)
	Men	16 (6%)	164 (59%)	83 (30%)	27 (10%)	28 (10%)	253 (92%)	192 (70%)	229 (83%)
** **	Total*	75 (7%)	677 (67%)	375 (37%)	617 (62%)	141 (14%)	825 (82%)	617 (62%)	502 (50%)

*Number of patients in study, 1004.

() = percentage.

Early sensory relearning was given to 342 patients (34%), traditional sensory re-education to 139 (14%) and non-structural exercises to 196 (20%). Three-hundred and twenty-seven patients (33%) received no sensory relearning. We found no difference regarding the extent to which patients with partial or total nerve injuries received sensory relearning (p = 0.711). Sensory assessments with static two-point discrimination (S2PD) and Semmes-Weinstein monofilaments (SWM) were documented in only 75 of the 1004 patients (in 7% of the male and 9% of the female patients).

**Fig 6:**
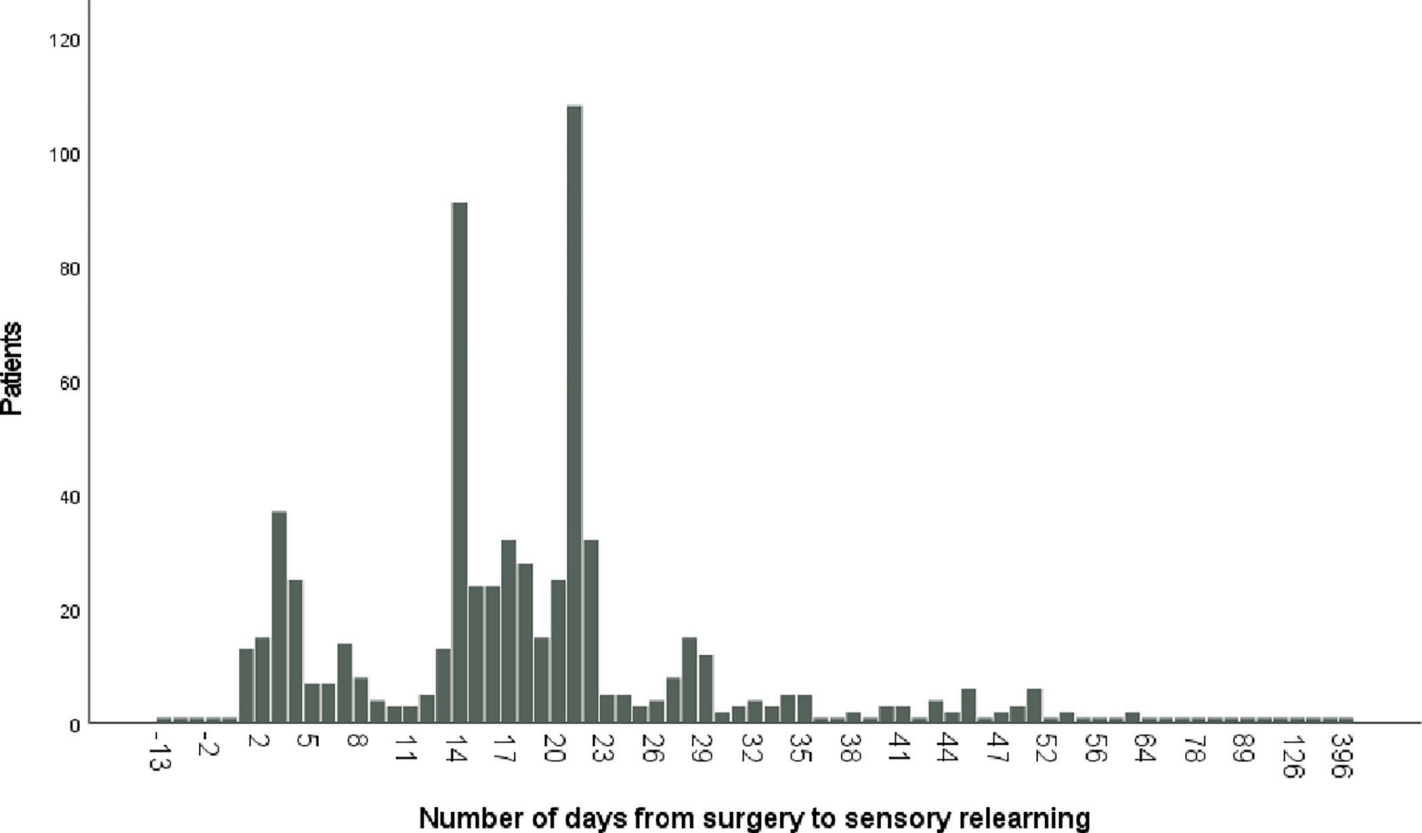
Bar chart presenting time from surgery to sensory relearning.

The median time from surgery to start of sensory relearning was 18 days (IQR 7) (range -13-396) for the entire population (the negative value represents pre-operative sensory relearning in four patients). There were no significant differences between female (17 days, IQR 7, range -6-293) and male patients (18 days, IQR 9, range -13-396). No significant differences were noted between patients with isolated nerve injuries and those with a concomitant flexor tendon injury (p = 0.321). Sensory relearning was most often initiated at 14 (n = 91, 9%) or 21 (n = 108, 11%) days after surgery (Fig 6).

## Discussion

In this population-based study on adult patients surgically treated for digital nerve injuries we present the incidence, injury mechanisms and details on rehabilitation interventions. Our results show small differences in injury mechanism, time to surgery, care and rehabilitation between patient groups (sex, concomitant tendon injury and type of injury). In addition, our results show that the timing of surgical repair and use of rehabilitation resources are often adapted to the health care system rather than tailored to individual patient needs.

Our study shows an incidence rate of digital nerve injuries in line with a previous study on a Swedish population [1]. The population in our study was larger than theirs but did not include patients <18 years of age. Our findings that male patients were more likely to sustain a digital nerve injury are supported by other authors. However, the proportion of male patients in our population (62%) was lower than in previous studies (74–78%) [1, 18, 19]. Two large European register studies on peripheral nerve injuries support our results, that young men are more likely to be affected by peripheral nerve injuries [20, 21]. The studies were conducted in Germany, where living terms and access to health care resemble Swedish conditions.

The consequences of a finger nerve injury are often permanent. Thus, understanding injury mechanisms is vital for optimal injury prevention. Our finding that a large proportion of injuries was due to sharp cuts is supported by Manninen et al. [22]. These authors reported that 39% of the injuries in their study were caused by knives and 14% by glass. In contrast, Chang et al. found glass to be the most common injury mechanism (36%), followed by knives (24%) [23]. While it might seem like an unlikely injury, cutting yourself with a knife when splitting an avocado was common in our study. Other studies report that this is a growing cause of injury [24, 25]. A US study reported that a large proportion of peripheral nerve injuries originate from sports and recreational activities [26], which we did not find in our study. The high number of sharp cuts in our study could reflect an urban city population not working with heavy machinery. However, we excluded patients with amputations, fractures and injuries requiring microsurgical repair, which likely underestimates the rate of digital nerve injuries in crush and lacerating injuries. Our data show that men had a higher proportion of concomitant tendon injuries compared to women, as supported by other studies [1, 22]. Differences in behaviour or activities between the sexes may explain different injury patterns.

Median sick leave in our study population was shorter than in a previous Swedish study reporting between 59–64 days [1]. These findings could reflect a change from industrial work to work in the service sector, much like the setting in our study. Differences in sick leave after injuries must be expected in different parts of the world based on differences in social security systems, making comparisons between studies difficult. Our findings can therefore only be compared to countries with sick leave compensation systems similar to ours. As expected, sick leave was longer in patients with concomitant flexor tendon injuries than isolated nerve injuries. These differences in sick leave are probably explained by the restrictions in load that are usually recommended after flexor tendon injuries.

A previous study showed a 20% lower risk for hand injury on Tuesdays [27], whereas other studies found no differences between weekdays [28]. In our study, digital nerve injuries were distributed equally over all months and days of the week. Aman et al report a higher incidence of peripheral nerve injuries in July [21]. However, surgery in our study, was most often conducted from Monday to Friday. Our clinic is not organized to treat flexor tendon and digital nerve injuries surgically during the weekends, indicating that resources were used at the convenience of the healthcare system rather than adjusted to the patient’s condition. The timing of surgery is considered an important factor for optimal return of sensation [29]. A recent publication suggested that acute repair should be performed within 14 days of injury [30]. In our study 124 patients (12%) waited over 7 days for nerve repair and 50 patients (5%) waited more than 14 days. The timing of surgery in relation to the final clinical outcome warrants further investigation. Our median time from surgery to sensory relearning was most often timed with an appointment scheduled for removal of the cast or sutures and not optimised to the timing for sensory interventions as described by Rosen and Lundborg [31, 32] which further reveals priority given to the health care process rather than individual patient needs.

Health care use in our study showed no sizeable differences between women and men. However, previous studies have indicated higher health care consumption among women [33]. The present study shows that women were more likely to be operated on earlier despite less severe injuries with fewer tendon injuries. Surprisingly, for some patients, the number of visits to our hand surgery clinic after an isolated digital nerve injury could be as high as 18 post-operative visits to an occupational therapist and 14 to a nurse. In contrast, other patients had no visits. The high number of visits could be indicative of a high frequency of complications. Yet, our study did not show more complications than previous studies [4].

Compared to previous studies, our study shows that a higher proportion of patients received some form of rehabilitation after digital nerve injury [1, 4, 27]. Few studies have described the content of rehabilitation interventions after a digital nerve injury [5–7]. In our study 33% of the patients did not receive sensory relearning despite having an injury to a sensory nerve. This might be due to no referral, failed visits by the patients, and in cases with concomitant tendon injury this might have been deprioritised or missed. Studies on major nerve injuries showed better tactile gnosis and discriminative touch when patients received early sensory relearning [9, 31]. We suspect that sensory relearning may benefit the results after digital nerve injuries [4, 34] and previous studies suggest a critical period for sensory relearning after nerve repair. Studies on major nerve injuries suggests initiation of sensory relearning before regeneration reaches the hand [9, 31]. If knowledge from those studies is applicable to digital nerve injuries sensory relearning should start even earlier due to the more distal location of the injury.

Our study found a documented sensory evaluation in only 7% of patients, making it difficult to conclude to what extent different rehabilitation interventions affect the outcomes after these injuries. Lack of sensory examinations could reflect the absence of standardised assessment tools. The Rosen score is the standard instrument for evaluating functional outcomes after major nerve injuries [35, 36]. It has been shown that after digital nerve injuries, the S2PD test is the most commonly used assessment tool [4]. The S2PD has been criticised because sensation and hand function require complex and integrated functions [37]. Examination with S2PD alone does not capture all aspects of the difficulties seen after these injuries. Yet, sensory function is essential to hand function and sensory assessment is crucial in the final evaluation of treatment outcomes. Pain and cold intolerance have been reported in many patients after nerve injury and repair [38]. One study reported that as much as 79% of patients suffered from cold intolerance [1]. We observed that very few patients received rehabilitation for pain and cold intolerance, but this does not rule out the possibility that patients may have had these problems.

The postoperative care after a digital nerve injury differs regarding content and follow-up. There is currently a lack of national guidelines in Sweden for the postoperative care of patients with digital nerve injuries. For many other diagnoses, national guidelines have been developed [39, 40] that may minimise differences in health care use. National guidelines may also reduce health inequalities influenced by education and socio-economic differences [41].

A systematic description of the working conditions of patients would be valuable in assessing potential differences between professions in terms of the safety of workers, treatment choices, sick leave or prognosis. During our study analysis, we searched the literature for a suitable classification system for demanded workload on the hands but found it difficult to identify a reasonable standard. Instead, we classified professions into categories according to ergonomic models [42]. Our choice of groups and their contents can rightfully be questioned. A validated and internationally accepted classification of the requirements of dexterity, grip strength, tolerance to cold environments and other aspects of hand function in different occupations would enable better comparisons between socio-economic groups, regions and countries.

### Strengths and limitations

A strength of this study is that it was undertaken in close collaboration between surgeons and occupational therapists. Another strength was the large number of digital nerve injuries. A limitation of the study is that some cases of digital nerve injuries may have been missed despite efforts to collect comprehensive data. Patients were excluded if injured and operated on in Stockholm but living outside the region. This approach allowed us to calculate the incidence rate in the population and include all post-operative health care interventions. Retrospective studies typically depend on already available data, initially not collected for research. It can be incomplete or inaccurate and potentially affect the validity [43]. In our study based on medical records and registry data, some information was missing. This includes details about dexterity, if the injury was work related, education and socio-economic status, timing and details about complications such as neuropathic pain. Another limitation is that our study population mainly represents an urban population, and our findings may not be generalisable to a rural population.

## Conclusion

This large Swedish descriptive study on digital nerve injuries includes data from the entire process of care relevant to both surgeons and rehabilitation professionals. The incidence of digital nerve injuries in this study was comparable to previous publications. There was a large individual variation in consumption of health care resources. Surprisingly few patients were assessed regarding sensory outcome and only two thirds of patients had received rehabilitation aiming at nerve reinnervation. There is a need for agreement on standardised assessments and care after digital nerve injury. Furter research is needed to evaluate outcome and to improve rehabilitation techniques. Treatment results are not reported in this study, and future studies are needed to investigate long term results after digital nerve injury, including different aspects of rehabilitation, hand function and sensation.
